# Cold Acclimation and Deacclimation of Two Garden Rose Cultivars Under Controlled Daylength and Temperature

**DOI:** 10.3389/fpls.2020.00327

**Published:** 2020-03-24

**Authors:** Lin Ouyang, Leen Leus, Ellen De Keyser, Marie-Christine Van Labeke

**Affiliations:** ^1^Institute of Urban Agriculture, Chinese Academy of Agricultural Sciences, Chengdu, China; ^2^Department of Plants and Crops, Ghent University, Ghent, Belgium; ^3^Plant Sciences Unit, Flanders Research Institute for Agriculture, Fisheries and Food (ILVO), Melle, Belgium

**Keywords:** cold stress, LT_50_, stem water content, bud dormancy, dehydrins, carbohydrate metabolism, gene expression

## Abstract

Low temperature stress is an important abiotic stress for garden roses in northern regions. Two garden rose cultivars (‘Dagmar Hastrup’ and ‘Chandos Beauty’) were selected to study the role of dehydrin and of carbohydrate metabolism during cold acclimation and deacclimation under the controlled daylength and temperature. The presence of bud dormancy was also observed as this could prevent budburst during warm spells. Both cultivars showed a similar changing pattern of cold acclimation and deacclimation and did not differ in their lowest LT_50_ values. *Dehydrin* (*RhDHN5*) was up-regulated by low temperatures and not by dehydration stress as the stem water content remained stable during the treatments. Total soluble sugars accumulated with a transient up-regulation of *RhBAM3* (a key gene in starch hydrolysis) for ‘Dagmar Hastrup’ at 2°C and a strong expression under both 2 and −3°C for ‘Chandos Beauty’. At 2 and −3°C, raffinose and stachyose strongly accumulated though the up-regulation of *RhRS6* and *RhGK* differed in the cultivars. Although similar cold hardiness levels were reached, carbohydrate metabolism in response to cold stress is different in the two cultivars. Increasing the temperature after a cold period resulted in fast deacclimation as found by the downregulation of *RhDHN5* and *RhBAM3*, the decrease of raffinose and stachyose. Bud endodormancy was hardly present in both cultivars.

## Introduction

In order to withstand freezing temperatures in mid-winter, woody perennials will develop low temperature tolerance during autumn, also called cold acclimation. Cold acclimation is strongly influenced by environmental triggers and is mainly induced by short days and/or lower temperatures. After reaching maximum levels of cold hardiness in winter also called mid-winter hardiness, increasing temperatures toward spring induce the deacclimation process leading to a reduction or loss of cold hardiness. Most woody plants also develop physiological bud dormancy or endodormancy in autumn, a state of temporal growth suspension within the meristem (Rohde and Bhalerao, 2007). However, low temperatures can induce cold acclimation without inducing dormancy (Kalberer et al., 2006). In woody plants native to temperate and boreal zones, bud dormancy and cold acclimation are governed through a complex but precise regulation of genes (Welling and Palva, 2006; Ruttink et al., 2007; Wisniewski et al., 2015). These two processes can be induced by low, non-freezing temperatures and/or short daylength although these environmental triggers are species dependent (Li et al., 2003; Heide, 2008; Wisniewski et al., 2014). The presence of endodormancy prevents regrowth and concomitant deacclimation during warm spells in winter (Kalberer et al., 2006; Wisniewski et al., 2018).

Cold acclimation and deacclimation are related to changes in cellular water status (Pagter and Arora, 2013). During cold acclimation, tissue dehydration has been detected in many woody plants as the decrease of free water resulting in higher tolerance to mechanical stress caused by extracellular ice formation (Li et al., 2002; Améglio et al., 2003). In contrast, deacclimation is associated with increasing tissue water content in perennials (Pagter et al., 2011a).

Recently, Wisniewski et al. (2018) reviewed the molecular control of cold tolerance in trees. Cold acclimation involves a transcriptomic reprogramming where the C-repeat/DREB binding factor (*CBF*) dependent pathway plays a central role (Shi et al., 2018). This has been largely investigated in *Arabidopsis thaliana* and is also identified in poplar, blueberry, apple and peach (Wisniewski et al., 2014). A large set of cold responsive (*COR*) genes can be induced via the *CBF*-dependent pathway and regulates the accumulation of cryoprotective proteins and soluble sugars, thus protecting membranes and reducing cellular dehydration (Chinnusamy et al., 2007).

Dehydrins are the D-11 subgroup of late embryogenesis abundant proteins (LEA) which are encoded by *COR*. The protein sequence is highly hydrophilic and they are involved in the plants’ protective reactions to cellular dehydration and they have antifreeze and/or cryoprotective properties (Close, 1997; Wisniewski et al., 1999; Hanin et al., 2011). The accumulation of dehydrins is frequently observed in woody plants during cold acclimation (Wisniewski et al., 1996; Close, 1997). Seasonal patterns of dehydrin proteins and transcripts are present in woody plants and a decline in both protein quantification and gene expression in spring has been associated with deacclimation (Artlip et al., 1997; Kontunen-Soppela et al., 2000; Arora et al., 2004). *RhDHN5* and *RhDHN6* are isolated based on the dehydrin (*ppdhn1*) in peach (*Prunus persica*) which has higher transcript levels in the bark tissue of both evergreen and deciduous peach during autumn and early winter (Artlip et al., 1997). *RhDHN5* and *RhDHN6* also present a seasonal expression pattern showing an up-regulation in November–January and down-regulation from February to April (Ouyang et al., 2019a).

It is assumed that dehydrins interact with sucrose by forming stable glasses (Wolkers et al., 2001) and are regulated by sucrose during cold acclimation (Rekarte-Cowie et al., 2008). The accumulation of soluble carbohydrates together with the accumulation of dehydrins may help the plants to develop their maximum levels of cold hardiness (Trischuk et al., 2014). Soluble carbohydrates act as compatible solutes (osmoregulators/osmolytes) to stabilize cellular osmotic potential to lower the freezing point and prevent the formation of intracellular ice crystals (Guy, 1990; Xin and Browse, 2000; Li et al., 2004). Their accumulation under low temperature has also a role in scavenging of reactive oxygen species and they act as signaling molecules (Welling and Palva, 2006; Theocharis et al., 2012). Degradation of starch and accumulation of soluble sugars especially sucrose and raffinose family of oligosaccharides (RFOs) are frequently reported during cold acclimation in *Arabidopsis* and several woody plants (Palonen, 1999; Palonen and Junttila, 1999; Guy et al., 2008; Pagter et al., 2008). Re-synthesis of starch at regrowth during spring accompanied by loss of soluble sugars was observed in woody plants during deacclimation (Morin et al., 2007; Pagter and Arora, 2013). Genes encoding galactinol synthase and BAM (a key enzyme in starch degradation) are up-regulated by the CBF regulon during cold acclimation, resulting in higher soluble sugar levels (Fowler and Thomashow, 2002; Wisniewski et al., 2014). Likewise, genes encoding SPS and INV in sucrose metabolism and raffinose synthase (RS) in RFOs biosynthesis change in abundance during cold acclimation (Usadel et al., 2008; Yue et al., 2015).

Despite the economic importance of roses in gardens and landscaping, and breeding efforts to develop roses with improved hardiness, hardly any reports on bud dormancy or factors affecting cold acclimation can be found. Observations under field conditions showed that dehydrins and carbohydrate metabolism are associated with cold hardiness in garden roses (Ouyang et al., 2019a). However, seasonal observations are subject to fluctuating temperatures, which result in acclimation/deacclimation/reacclimation events, making it difficult to distinguish the molecular/metabolic changes associated with cold acclimation. Furthermore, hardly any literature reports on whether the dormant state of garden roses during winter is linked to environmental parameters (ecodormancy) or if a true endodormancy is established.

In the present study, we aimed to characterize basic differences of cold acclimation in two cultivars with distinct genetic background and different mid-winter hardiness. ‘Dagmar Hastrup’ (diploid, Hybrid Rugosa), introduced in 1914, is closely related to the species *Rosa rugosa*. In contrast, ‘Chandos Beauty’ (tetraploid, Hybrid Tea), introduced in 2005, has a complicated genetic background (including ±10 rose species) reflecting the development of modern rose cultivars. In a 3-year field study including 17 different cultivars/species, ‘Dagmar Hastrup’ belonged to the most cold-hardy group while ‘Chandos Beauty’ belonged to the most cold-susceptible group (Ouyang et al., 2019b). Furthermore ‘Dagmar Hastrup’ developed a higher level of mid-winter hardiness (determined by LT_50_) than ‘Chandos Beauty’ in a field study (Ouyang et al., 2019a). This contrast in acquiring cold hardiness predicts different strategies to face up to the challenges imposed by freezing temperatures. To test this hypothesis, we simulated the progression of cold acclimation in natural conditions and analyzed dehydration and carbohydrate dynamics under a controlled drop in temperature and photoperiod (acclimation) followed by a subsequent increase (deacclimation) using gene expression and metabolite analysis. Further, we investigated the presence of bud dormancy as this might also influence the process of cold acclimation and may be different in both cultivars. Through this approach, we aim to further clarify the role of dehydrins and carbohydrate metabolism in cold hardiness in garden roses.

## Materials and Methods

### Plant Material

Two garden roses, ‘Dagmar Hastrup’ (diploid, Hybrid Rugosa, more cold-hardy; coldest USDA zone 3b) and ‘Chandos Beauty’ (tetraploid, Hybrid Tea, less cold-hardy, coldest USDA zone 6b), were selected for this study. Plants of each cultivar were clonally propagated and obtained from Pheno Geno Roses BV (Eindhoven, Netherlands).

For the bud dormancy experiment (2017–2018), current year stems of field-grown roses (51° 0′ N, 3° 48′ E, Melle, Belgium) were used. Roses were planted in a double row, with 30 plants per cultivar per row. Plants had been established 3 years before sampling.

For the controlled acclimation and deacclimation experiment, 1-year-old container-grown plants (2 L pots, 60 plants/cultivar), still actively growing, were obtained at the end of October 2016. Upon arrival, plants were kept in the greenhouse to maintain active growth until the start of the experiment (minimum air temperatures 5°C). During the daytime plants were supplemented with 100 μmol m^–2^ s^–1^ PPFD at canopy when the outdoor light intensity was lower than 75 W m^–2^ (600 W SON-T lamps, Philips, Eindhoven, Netherlands).

### Experiments

#### Experiment 1: Bud Dormancy

From the field grown plants, a randomly chosen set of 30 current-year stems was collected from both cultivars on September 18, 2017, October 20, 2017, November 15, 2017, December 20, 2017, January 18, 2018, and February 17, 2018, respectively. Bud dormancy was measured by a single node cutting test (Arias and Crabbé, 1975). Nodes from the second and third apical position of the chosen 30 stems of each cultivar were cut in single-node cuttings (±1-cm stem sections) (*n* = 60). The sections were inserted into a peat substrate in trays and were placed in a growth chamber at 20 ± 2°C, relative humidity ± 70%, daylength 16 h and light intensity 100 μmol m^–1^ s^–1^ (Plasma lamps, Gavita International, Aalsmeer, Netherlands). Bud break (BBCH scale 07 when scale tips dispersed along the bud axis) (Meier et al., 2009) was recorded three times per week. The percentage of bud dormancy was calculated after 30 days of observation. Buds that failed to break were assigned a time of 30 days. The mean time (days) to bud break (MTB) was calculated based on the formula below.

$$M\,T\,B=\frac{\begin{array}{c} \sum \{\,\left(n\,u\,m\,b\,e\,r\,o\,f\,n\,e\,w\,b\,r\,e\,a\,k\,i\,n\,g\,b\,u\,d\,s\right)\times d\,a\,y\,\}+\\ n\,u\,m\,b\,e\,r\,o\,f\,n\,o\,n\,b\,r\,e\,a\,k\,i\,n\,g\,b\,u\,d\,s\times 30 \end{array}}{t\,o\,t\,a\,l\,n\,u\,m\,b\,e\,r\,o\,f\,t\,e\,s\,t\,e\,d\,b\,u\,d\,s}$$

#### Experiment 2: Controlled Acclimation and Deacclimation

The experimental design was a controlled acclimation and deacclimation protocol using 60 potted multi-branched roses for each cultivar (Supplementary Figure S1). This protocol is based on Palonen et al. (2000) and modified with respect to the results of our previous field study (Ouyang et al., 2019a). The branches per pot were selected randomly and used for a one-date sampling during the whole acclimation-deacclimation treatment. The experiment started on December 19, 2016 and ended February 27, 2017. A temperature of 20/18°C (day/night) and photoperiod of 16 h (SON-T high pressure lamps, 100 μmol⋅m^–2^⋅s^–1^ PPFD at plant level) was provided for the plants under greenhouse conditions for 3 weeks until 19 December, followed by a decrease of temperature (13/6°C) and daylength (8 h) for 4 weeks (20 December to 16 January). Besides the sampled pots, other plants were placed in a climate chamber for 2 weeks at 2°C (17 January to 30 January) followed by 2 weeks at sub-zero temperatures (−3°C) (31 January to 13 February). The photoperiod remained at 8 h but the light intensity was reduced to 5–10 μmol⋅m^–2^⋅s^–1^ as provided by fluorescent lamps (Philip Master TL-D 36W/830 Reflux, Philips, Eindhoven, Netherlands). Finally, a deacclimation phase was simulated in a greenhouse at 100 μmol⋅m^–2^⋅s^–1^ PPFD with the remaining plants 1 week at 20/18°C with a photoperiod of 8 h (14 February to 20 February) followed by 1 week at 20/18°C with a photoperiod extended to 14 h (21 February to 27 February).

Current-year stems were sampled for the determination of cold hardiness, stem water content, and carbohydrate content (four biological replicates per cultivar per analysis) on 19 December, 2 January, 16 January, 23 January, 30 January, 6 February, 13 February, 20 February, and 27 February (9 time points). For the gene expression analysis, stem tissue was sampled (three biological replicates per cultivar) on 19 December, 20 December, 2 January, 16 January, 17 January, 23 January, 30 January, 31 January, 6 February, 13 February, 14 February, 20 February, and 27 February (13 time points). Four extra sampling time points (20 December, 17 January, 31 January, and 14 February) were included for gene expression analysis, as a transient expression may happen when environmental conditions change. For both gene expression and carbohydrate analysis, each replicate was a balanced mix of the apical, middle, and basal part of one individual stem. For the determination of cold hardiness and water content, samples were kept on ice and immediately transferred to the laboratory for analysis. Samples for carbohydrate analysis and gene expression were flash frozen in liquid nitrogen and stored at −80°C until analysis.

### Determination of Cold Hardiness

Cold hardiness levels were determined by means of LT_50_ (the temperature causing 50% of index of injury) in a controlled freezing test for each cultivar (*n* = 4). Internodal stem segments (0.5-cm-long) were taken from the middle part of the current-year stem, then rinsed under distilled water for 10 s and placed in 2 mL Eppendorf microcentrifuge tubes (Eppendorf, Hamburg, Germany) with 0.5 mL distilled water and a few grains of sand (VWR International, Leuven, Belgium). Within each repetition, one stem segment was maintained at a reference temperature of 4°C as control. Stem segments were placed in a cryostat of Polystat 37 (Fisher Scientific, Merelbeke, Belgium) from 0°C to seven target temperatures (−5, −10, −15, −20, −25, −30, and −35°C) at a cooling rate of 6°C h^–1^ (0.1°C min^–1^). Meanwhile, one segment was placed at −80°C for 5 h. After thawing overnight at 4°C, all the segments were incubated in 10 mL of 10 mM boric acid (H_3_BO_3_) and 0.002% Triton-X at room temperature for 20 h. EL was measured before and after autoclaving at 120°C for 30 min using WTW Inolab conductivity meter Level 1 (WTW GmbH & Co. KG, Weinheim, Germany) with a TetraCon 325 conductivity cell. Index of Injury (*I*_*t*_) based on EL values was calculated according to Flint et al. (1967) and transformed into the adjusted *I*_*t*_ value taking into account the *I*_*t*_ at −80°C (100% injury value) (Lim et al., 1998). The LT_50_ was calculated from the injury versus temperature plot by means of logistic regression.

### Stem Water Content

Fresh weight (FW) of 5–6 cm segments of each cultivar from the median part of the stem was determined, followed by drying at 80°C for 48 h to determine the dry weight (DW) (*n* = 4). Stem water content (g H_2_O g^–1^ DW) was calculated on a dry weight basis as [(FW-DW)/DW].

### Analysis of Carbohydrates

Tissue samples of stems were ground in liquid nitrogen with a mill (IKA^®^ A11 Basic Analytical Mill, Staufen, Germany). Soluble carbohydrates were analyzed as described by Ouyang et al. (2019a) for each cultivar (*n* = 4). In short, soluble carbohydrates were extracted in 80% ethanol and quantified by high performance anion-exchange chromatography with pulsed amperometric detection (ACQUITY UPLC H-Class, Waters, Milford, MA, United States). Starch, expressed as glucose equivalents after acid hydrolysis, was determined spectrophotometrically at 340 nm by the enzymatic reduction of NADP^+^ (UV-VIS, Biotek Uvikon XL, Bad Friedrichshall, Germany) with a HXK/glucose-6-phosphate dehydrogenase assay.

### RNA Extraction and Reverse Transcription

Tissue samples of stems were ground in liquid nitrogen using a mortar and pestle. RNA was extracted from 100 mg of ground tissue sample using a CTAB protocol for each cultivar (*n* = 3). RNA quality was assessed with the NanoDrop (ND-1000) spectrophotometer (Isogen Life Science, Utrecht, Netherlands) (Supplementary Table S1). RNA integrity was determined by the Experion Automated Electrophoresis System and RNA StdSens Chips (Bio-Rad Laboratories N.V., Temse, Belgium) on a random selection of over 10% of the total sample numbers across both cultivars and all sampling points (Supplementary Figures S2, S3). RNA samples were converted to single-stranded cDNA using the iScript cDNA Synthesis Kit (Bio-Rad Laboratories N.V., Temse, Belgium) starting from 600 ng of RNA. Detailed protocols were as described in Luypaert et al. (2017).

### Gene Isolation

Coding sequences of *RhSPS1* (involved in sucrose metabolism), and *RhHXK1* and *RhFRK4* (involved in hexose catabolism) were newly obtained (Supplementary Table S2) and the method is as described in Ouyang et al. (2019a). BLASTx (Altschul et al., 1997) was used to confirm fragment identity (Supplementary Table S3). In addition to these four sequences, eight sequences obtained previously in Ouyang et al. (2019a) were also studied here: seven key genes associated in carbohydrate metabolism (*RhBAM3* and *RhDPE2* in starch catabolism, *RhSUS* and *RhINV2* in sucrose metabolism; *RhMIPS* in myo-inositol synthesis pathway, and *RhGK* and *RhRS6* in the raffinose synthesis pathway) and *RhDHN5* (dehydrin gene) (Supplementary Table S2). Given the similar up-regulation pattern of *RhDHN5* and *RhDHN6* and their link to cold hardiness (Ouyang et al., 2019a), we limited further research to *RhDHN5.*

### Gene Expression

RT-qPCR primers for *RhSPS1*, *RhHXK1*, and *RhFRK4* were designed using Primer3Plus software (Untergasser et al., 2007) (Supplementary Table S4). RT-qPCR primers for all the other genes (including two reference genes) were designed by Ouyang et al. (2019a) (Supplementary Table S4). Gene specific amplification efficiencies were determined by LinRegPCR (Ruijter et al., 2009) (Supplementary Table S4). RT-qPCR analysis was performed as described in Ouyang et al. (2019a) using cDNA synthesized from RNA samples of stem tissues. A normalization factor based on two validated reference genes (*PGK and RPS18c*; *M*-value = 0.391; CV-value = 0.137) was used for calculation of calibrated normalized relative quantities (CNRQ) using qbase+ software (Biogazelle, Ghent, Belgium) (Hellemans et al., 2007). CNRQ values were exported to Microsoft Excel. Biological replicates were averaged geometrically.

### Statistical Analysis

Homoscedasticity of all the data was checked by Levene’s test (*P* ≥ 0.01). If homoscedasticity of data was fulfilled, data were analyzed using ANOVA with Scheffé’s *post hoc* test (*P* = 0.05). If homoscedasticity of data failed the Levene’s test, data were analyzed using one-way ANOVA with Games–Howell’s *post hoc* test for physiological data (LT_50_, stem water content and carbohydrate concentration) and using Kruskal–Wallis test (*P* = 0.05) with Bonferroni correction for multiple tests for gene expression data. Calibrated normalized relative quantity (CNRQ) values of gene expression data were log-transformed. Correlations between LT_50_ and physiological data were analyzed by Pearson’s two-tailed test (*P* = 0.05), while correlations between LT_50_ and gene expression were analyzed by Spearman’s two-tailed test (*P* = 0.05) excluding the data of the transcript levels on climate changing points (20 December, 17 January, 31 January, and 14 February). If *P* > 0.05, there is no significant correlation; if *P* < 0.05, there is a significant correlation; if *P* < 0.01, there is a moderately strong correlation; and if *P* < 0.001, there is a very strong correlation. Statistical analyses were performed in SPSS Statistics (version 24) and all figures were made in SigmaPlot (version 13.0). Gene expression graphs are based on non-log transformed data.

## Results

### Seasonal Changes in Bud Dormancy

**FIGURE 1:**
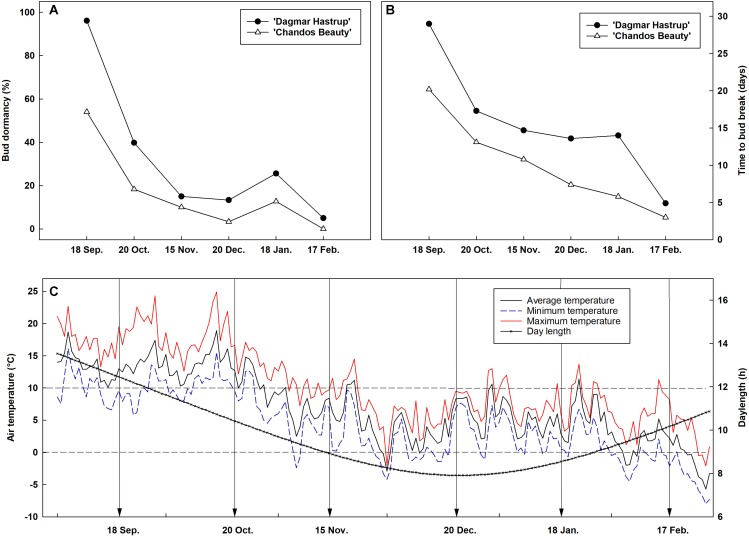
Seasonal evolution of bud dormancy **(A)** and mean time to bud break **(B)** from 60 single-node cuttings in the apical zone of ‘Chandos Beauty’ and ‘Dagmar Hastrup’ from September 2017 to February 2018 with the corresponding air temperature and daylength conditions **(C)** in the field. The arrow in **(C)** indicates the sampling time points.

Bud dormancy followed a similar seasonal dynamic for both cultivars, although a deeper dormancy level was found for ‘Dagmar Hastrup’ (Figure 1). For ‘Dagmar Hastrup’, most buds were dormant on 18 September (96.1%) (Figure 1A). However, cold requirements were low, only 39.8% of buds were dormant on 20 October when the average temperature was still above 10°C (Figures 1A,C). Bud dormancy decreased further to 15% on 15 November when average temperature dropped below 10°C for 21 days and even below 7°C for 10 days and daylength decreased below 10 h (Figures 1A,C). For ‘Chandos Beauty’, only 54% of the buds were dormant on 18 September, and dormancy released in 80% of the buds from October on. The mean time to bud break was highest in September and reached 29 days for ‘Dagmar Hastrup’ and 20 days for ‘Chandos Beauty.’ From October till February, the mean time to bud break decreased sharply for both cultivars (Figure 1B). From February on, the average temperature decreased below 5°C and even freezing temperatures occurred while the daylength increased from 9 to 10 h, bud break took only 3 days for ‘Chandos Beauty’ and 5 days for ‘Dagmar Hastrup’ on 17 February (Figure 1B).

### Change of Cold Hardiness and Stem Water Content Under Controlled Conditions

**FIGURE 2:**
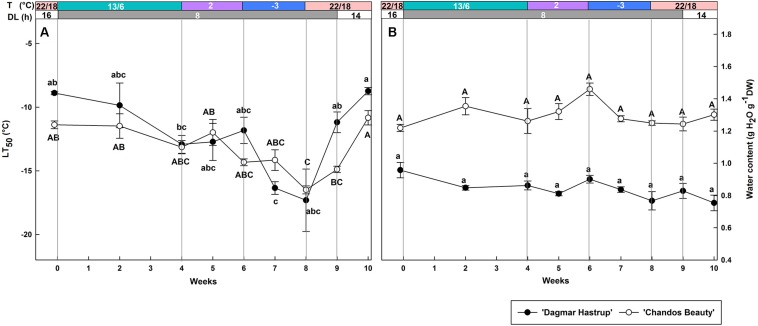
Changes in LT_50_ **(A)** and stem water content **(B)** for ‘Dagmar Hastrup’ and ‘Chandos Beauty.’ The strips above the figure indicate the provided temperature (T) and daylength (DL). Data of LT_50_ for ‘Dagmar Hastrup’ were analyzed using one-way ANOVA with Games–Howell’s *post hoc* test (*P* = 0.05). Data of LT_50_ for ‘Chandos Beauty’ and water content was analyzed using ANOVA with Scheffé’s *post hoc* test (*P* = 0.05). Different letters indicate significant differences between sampling dates for each cultivar. Value per cultivar per sampling date is mean ± SE (*n* = 4).

Both cultivars showed a similar changing pattern of cold hardiness levels during acclimation (weeks 0–8) and deacclimation (weeks 8–10) (Figure 2A). The LT_50_ of non-acclimated plants ranged from −8.9°C for ‘Dagmar Hastrup’ to −11.4°C for ‘Chandos Beauty.’ Decreasing the photoperiod to 8 h and lowering the temperature to 13/6°C reduced LT_50_ for both cultivars; decrease of the temperature to 2°C did not significantly decrease the LT_50_. A change to freezing temperatures at −3°C for 2 weeks (weeks 6–8) further decreased LT_50_ to significantly lower values of −16.5°C for both cultivars compared to week 0 (Figure 2A). Both cultivars reacted quickly to deacclimation temperatures of 22/18°C. ‘Dagmar Hastrup’ lost its cold hardiness after 1 week (week 9) under short daylength (8 h) while ‘Chandos Beauty’ was fully deacclimated after 2 weeks (week 10) when long days (14 h) were provided.

No significant variation in stem water content was found for both cultivars under changing temperatures and daylength (Figure 2B). ‘Chandos Beauty’ had, however, a higher stem water content ranging from 1.22 at the start to 1.46 g H_2_O g^–1^ DW after 10 weeks compared to ‘Dagmar Hastrup’ (0.96 g H_2_O g^–1^ DW at the start to 0.75 g H_2_O g^–1^ DW after 10 weeks).

### Carbohydrate Metabolism

**FIGURE 3:**
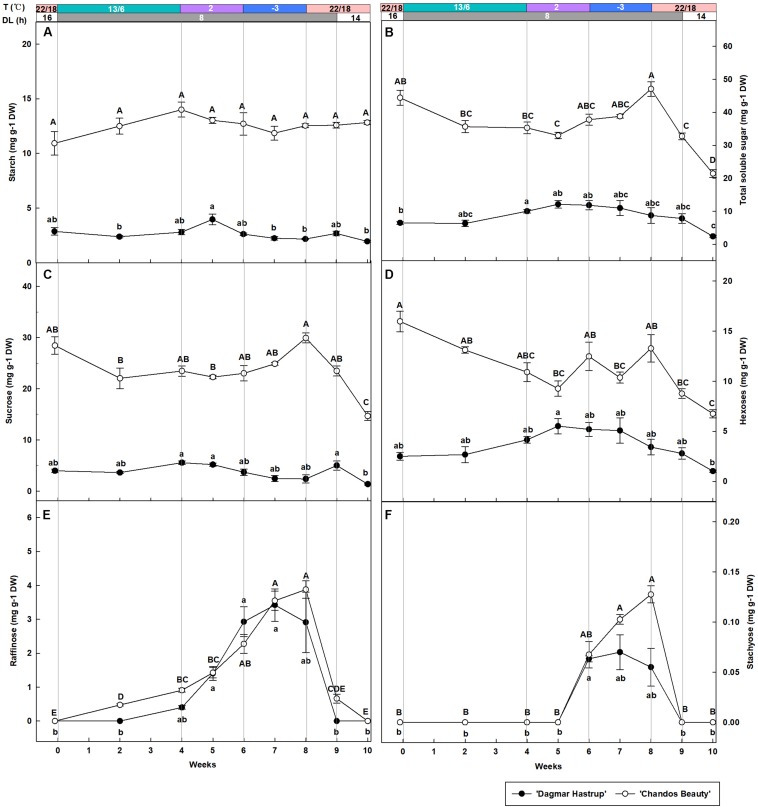
Changes in the content of starch **(A)**, total soluble sugars **(B)** sucrose **(C)**, hexoses (glucose + fructose) **(D)**, raffinose **(E)**, and stachyose **(F)** of ‘Dagmar Hastrup’ and ‘Chandos Beauty’ under controlled climate condition. The stripes above the figure indicate the provided temperature (T) and daylength (DL). Data of total soluble sugar: starch, raffinose, and stachyose were analyzed using one-way ANOVA with Games–Howell’s *post hoc* test (*P* = 0.05). Data of starch, sucrose and hexoses were analyzed using ANOVA with Scheffé’s *post hoc* test (*P* = 0.05). Different letters indicate significant differences between sampling dates for each cultivar. Value per cultivar per sampling date is mean ± SE (*n* = 4).

Overall, starch content was significantly lower for ‘Dagmar Hastrup’ than for ‘Chandos Beauty’ (*P* = 0.001, Figure 3A). For ‘Dagmar Hastrup,’ starch increased slightly reaching 4.0 mg g^–1^ DW after 1 week at 2°C (week 5), but dropped significantly at sub-zero temperatures by 45% to 2.2 mg g^–1^ DW (week 9) and remained low under deacclimation. In contrast starch levels remained relatively stable (10.9–13.0 mg g^–1^ DW) during cold acclimation and deacclimation in ‘Chandos Beauty.’

Total soluble sugars were significantly influenced by cold acclimation and deacclimation for both cultivars (Figure 3B). For ‘Dagmar Hastrup’, the content increased significantly by 54.4% from 6.5 to 10.0 mg g^–1^ DW during weeks 0–4 (13/6°C, 8 h) and remained stable at 2 and −3°C. The sugar content of ‘Dagmar Hastrup’ decreased by 72.6% during deacclimation (weeks 8–10, 22/18°C). For ‘Chandos Beauty,’ sugars decreased by 20.1% during weeks 0–4 (13/6°C, 8 h) and increased significantly by 33.5% during 2 and −3°C treatments reaching maximum of 47.1 mg g^–1^ DW at week 8 (−3°C, 8 h). During deacclimation (weeks 8–10; 22/18°C), sugar content decreased prominently by 54.5% for ‘Chandos Beauty’ to 21.4 mg g^–1^ DW.

Sucrose, hexoses (glucose + fructose), raffinose and stachyose were analyzed during acclimation and deacclimation, respectively (Figures 3C–F). Sucrose and hexose levels were significantly higher at the start for ‘Chandos Beauty’ than for ‘Dagmar Hastrup’ and remained higher during the experiment (Figures 3C,D). For ‘Dagmar Hastrup,’ sucrose hardly changed during the experiment (Figure 3C). Hexoses increased by 65.7% during weeks 0–4 (13/6°C, 8 h) and remained stable (between 3.4 and 5.5 mg g^–1^ DW) during 2 and −3°C treatments. Deacclimation resulted in decreasing hexose levels of ‘Dagmar Hastrup’ by 69.9% to 1.0 mg g^–1^ DW (Figure 3D). For ‘Chandos Beauty,’ the temperature drop to 13/6°C resulted in a decreasing of sucrose and hexoses by 17.5 and 31.7%, respectively (weeks 0–4). Further decrease of the temperature resulted in a non-significant increase by 27.5% for sucrose and fluctuating levels of hexose between 9.3 and 13.3 mg g^–1^ DW (weeks 4–8). Deacclimation temperatures (22/18°C) led to a significant decrease of both sucrose and hexoses of ‘Chandos Beauty’ by 50.0 and 49.1%, respectively (Figures 3C,D).

Both raffinose and stachyose content was strongly influenced by ambient temperatures for both cultivars (Figures 3E,F). A decrease to 2°C resulted in an increase of raffinose; this increase was further enhanced by sub-zero temperatures. For stachyose, no change was noted until 2 weeks at 2°C with the content of 0.06 mg g^–1^ DW for both cultivars. Here again, sub-zero temperatures further enhanced stachyose content for ‘Chandos Beauty’ but not for ‘Dagmar Hastrup’ (Figures 3E,F). For ‘Dagmar Hastrup,’ the raffinose and stachyose peaked at the end of week 7 at 3.4 and 0.07 mg g^–1^ DW, respectively. For ‘Chandos Beauty,’ the largest amount of raffinose (3.5 mg g^–1^ DW) and stachyose (0.13 mg g^–1^ DW) were found at the end of week 8.

Additionally, raffinose and stachyose correlated negatively (*P* < 0.01) with LT_50_ for both cultivars. For ‘Chandos Beauty,’ total soluble sugars and sucrose were negatively correlated with LT_50_. No correlations were found for hexoses (Table 1).

**TABLE 1:** Pearson’s correlation coefficients between LT_50_ and carbohydrate concentration, and Spearman’s correlation coefficients between LT_50_ and gene expression for ‘Dagmar Hastrup’ and ‘Chandos Beauty,’ *P < 0.05, **P < 0.01; ns, not significant.

Test items	LT_50_ of ‘Chandos Beauty’	LT_50_ of ‘Dagmar Hastrup’
Total soluble sugars	-0.44*	ns
Sucrose	−0.48**	ns
Raffinose	−0.67**	−0.56**
Stachyose	−0.63**	−0.53**
*RhDHN5*	−0.58**	−0.66**
*RhBAM3*	−0.53**	−0.55**
*RhDPE2*	ns	−0.64**
*RhSUS*	ns	0.62**
*RhFRK4*	ns	0.67**
*RhHXK1*	ns	0.53**
*RhMIPS*	ns	0.52**

### Expression Analysis of *RhDHN5*

**FIGURE 4:**
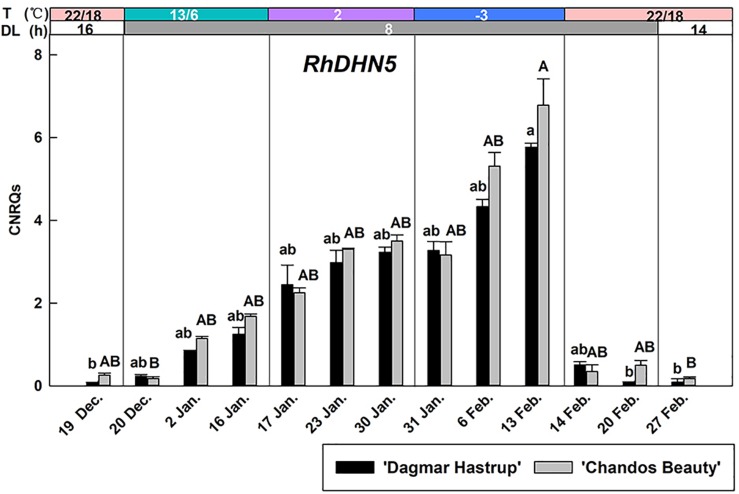
Expression of *RhDHN5* for ‘Dagmar Hastrup’ and ‘Chandos Beauty’ under a controlled climate. The stripes above the figure indicate provided temperature (T) and daylength (DL). Data were assessed by the Kruskal–Wallis test (*P* = 0.05). CNRQ (calibrated normalized relative quantity) per cultivar per sampling date is presented as geometric mean ± SE (*n* = 3). Different letters indicate significant differences between sampling dates for each cultivar.

The up-regulation of *RhDHN5* during acclimation was very similar for ‘Dagmar Hastrup’ and ‘Chandos Beauty’ (Figure 4). Decreasing the temperature to 13/6°C under short daylength gradually increased transcript levels. *RhDHN5* transcripts further accumulated by decreasing the temperature to 2°C and sub-zero temperatures. The highest expression levels (CNRQs around 6) were reached after 2 weeks at −3°C which induced a 25- and 76-fold change, respectively, compared to non-acclimated plants (22/18°C, 16 h, 19 December). Deacclimation (22/18°C with short days) leads to a strong and immediate decrease (after 24 h) in transcript levels with CNRQs below 1. Two weeks after the start of the deacclimation, transcript levels were similar to those of non-acclimated plants (19 December).

### Expression Analysis of Starch Metabolism-Related Genes

**FIGURE 5:**
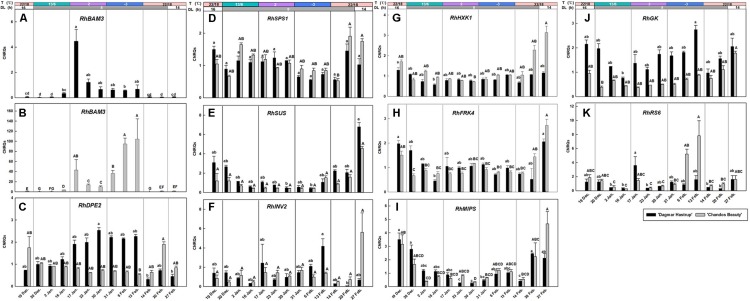
Expression of *RhBAM3* and *RhDPE2* associated with starch catabolism **(A–C)**, *RhSPS1*, *RhSUS*, and *RhINV2* in sucrose metabolism **(D–F)**, *RhHXK1* and *RhFRK4* in hexose catabolism **(G,H)**, *RhMIPS* in myo-inositol synthesis **(I)** and *RhGK* and *RhRS6* in RFOs synthesis **(J,K)**. The stripes above the figure indicate the temperature (T) and daylength (DL). Data of *RhBAM3*, *RhMIPS* and *RhRS6* were assessed by one-way ANOVA with Scheffé’s *post hoc* test (*P* = 0.05). Others were assessed by Kruskal–Wallis test (*P* = 0.05). CNRQ (calibrated normalized relative quantity) per cultivar per sampling date is presented as geometric mean ± SE (*n* = 3).

The expression of *RhBAM3* and *RhDPE2*, two genes involved in the starch catabolic pathway, are given (Figures 5A–C). A remarkable induction of *RhBAM3* was present in both cultivars when temperatures decreased to 2°C. As there is an important difference in the range of CNRQs levels of the two cultivars, results are presented separately (Figures 5A,B). For ‘Dagmar Hastrup,’ a transient higher expression was observed in *RhBAM3* 24 h after the ambient temperature dropped to 2°C. One week later, *RhBAM3* transcripts had decreased and remained relatively stable when temperatures further dropped below zero. During deacclimation (22/14°C), *RhBAM3* transcripts dropped significantly to nearly undetectable levels. For ‘Chandos Beauty,’ *RhBAM3* was transiently up-regulated 24 h after a change to 2°C and then decreased; the transcript levels increased again by a shift to −3°C and accumulated to their highest level in the following 2 weeks (Figure 5B). The expression of *RhDPE2* was not linked to *RhBAM3* (Figure 5C). For ‘Dagmar Hastrup,’ a higher transcript abundance of *RhDPE2* was seen when temperature was low (2 and −3°C, 17 January to 13 February). However, for ‘Chandos Beauty,’ low temperature did not induce expression of *RhDPE2* and higher transcript levels were present for non-acclimated plants (19 December) and after 1 week of deacclimation (22/14°C, 20 February).

### Expression Analysis of Sugar Metabolism Related Genes

The expression of *RhSPS1* decreased slightly from 1.05 to 0.67 under conditions of cold acclimation for ‘Dagmar Hastrup,’ while deacclimation enhanced its expression for both cultivars (Figure 5D).

For ‘Dagmar Hastrup’. *RhINV2* transcripts increased after 2 weeks at −3°C, while deacclimation downregulated the expression. For ‘Chandos Beauty,’ low temperatures (both 2 and −3°C) did not influence the expression levels of *RhINV2*, although 2 weeks of deacclimation increased fourfold of the expression compared to the expression at the end of −3°C treatment (Figure 5F).

The expression of *RhSUS*, *RhHXK1*, and *RhFRK4* was very similar for both cultivars (Figures 5E,G,H). Acclimation treatments resulted in low transcript levels while non-acclimated plants (start of the experiment) and deacclimated plants (end of the experiment) had higher transcript levels.

*RhMIPS* is involved in the biosynthesis of myo-inositol (Figure 5I). For both cultivars, the transcript levels of *RhMIPS* decreased during low temperature treatment from 13/6°C to −3°C. During deacclimation, when temperature raised to 20/18°C, transcript levels increased significantly for both cultivars after 1 week and were strongly induced for ‘Chandos Beauty’ when light provision shifted from short days to long days.

*RhGK* and *RhRS6* are involved in the biosynthesis of RFOs (Figures 5J,K). Shortening the photoperiod from 16 to 8 h at 13/6°C decreased the expression of *RhGK* from 2.15 to 0.75 for ‘Dagmar Hastrup.’ Lowering the temperature further to 2°C enhanced transcript levels, while further transcript accumulation was found after 2 weeks at −3°C (Figure 5J). For ‘Chandos Beauty,’ the transcript levels of *RhGK* were suppressed during cold acclimation and gradually increased during deacclimation (Figure 5J).

The up-regulation of *RhRS6* was cultivar dependent. For ‘Dagmar Hastrup,’ a transient increase was found 24 h after the temperature dropped from 13/6 to 2°C. A decrease to −3°C did not further affect the transcription of this gene (Figure 5K). For ‘Chandos Beauty,’ temperatures of 2°C did not influence transcript levels but freezing temperatures of −3°C resulted in an up-regulation after 1 week, with this up-regulation remaining high during the 2nd week at −3°C. A fast response to the deacclimation treatment (20/18°C) of *RhRS6* was found as the transcript levels reduced 24 h after the start of the treatment at 22/18°C (Figure 5K).

The correlation analyses between LT_50_ values and gene expression levels are given in Table 1. *RhDHN5* transcripts strongly correlated with LT_50_ for both cultivars (*P* < 0.01). In addition, for ‘Dagmar Hastrup,’ the expression of *RhDPE2* and *RhBAM3* (starch degradation) was negatively correlated with LT_50_. However, the transcript level of *RhSUS* (sucrose metabolism), *RhFRK* and *RhHXK* (hexoses catabolism), and *RhMIPS* (myo-inositol synthesis) are positively correlated with LT_50_. For ‘Chandos Beauty,’ only a negative correlation between LT_50_ and *RhBAM3* was present.

## Discussion

In an earlier field study we reported that ‘Dagmar Hastrup’ had a higher cold hardiness levels compared to ‘Chandos Beauty’ during the period November–January (Ouyang et al., 2019a). However, the understanding of environmental control on acclimation is hampered by the fluctuating environmental parameters in field studies. Also, it is not known if roses develop endodormancy, a process that might develop simultaneously with cold acclimation.

Our single node cutting experiment shows that endodormancy is present in the axillary buds of apical zones for both cultivars in September but less than 50% of the buds remain dormant in October. This indicates that roses have a low chilling demand, which is even lower for the less cold-hardy ‘Chandos Beauty’ as revealed also by a lower MTB (Figure 1). Rose buds are thus dominantly ecodormant (caused by environmental factors) during winter but differences in MTB indicate that cultivar differences in reaction to warm spells clearly exist till January. As both rose cultivars hardly showed bud endodormancy, warm spells may result in a fast bud break.

In this study, a controlled acclimation process did not result in a different level of cold acclimation between the cultivars (LT_50_ around −15°C, 8 weeks after the start of the cold acclimation treatment) although the more cold-hardy ‘Dagmar Hastrup’ reacts more strongly to the −3°C treatment (Figure 2A). The similar cold hardiness levels detected in these two cultivars may be due to a relative short period of low temperature treatment (2 weeks) and that therefore the ‘Dagmar Hastrup’ did not develop its maximum hardiness levels yet. Deacclimation occurred very rapidly and occurred sooner in ‘Dagmar Hastrup.’ A faster deacclimation than acclimation has also been observed in other woody plants and herbs (Gay and Eagles, 1991; Arora et al., 1992). Kalberer et al. (2007) found that the hardier *Rhododendron canadense* deacclimated faster than the less cold-hardy *Rhododendron prunifolium*. Likewise, it is found that the deacclimation level was greater in the hardier *Hydrangea* genotypes suggested that the deacclimation resistance is not correlated to the cold hardiness levels in mid-winter (Pagter et al., 2011b). Although a faster deacclimation may make the cultivar more susceptible to spring frost, the reacclimation capacity may also influence the resistance to these frost events. A rapid reacclimation was found in dehardened buds of three azalea genotypes within 1 day of exposure to a reacclimation regime (Kalberer et al., 2007) and the reacclimation capacity did not significantly associate with deacclimation resistance (Arora and Rowland, 2011). Therefore, reacclimation ability is also an important trait when coping with warm spells and needs to be further studied in roses.

Despite a similar level of cold acclimation between these rose cultivars, differences in physiological parameters and molecular patterns were found. Similar to our field results (Ouyang et al., 2019a), the more cold-hardy ‘Dagmar Hastrup’ had a stable and lower stem water content. A low tissue water content combined with a high amount of solutes (sugars and antifreeze proteins) slows down the speed of ice formation and migration of intracellular water to extracellular water (Gusta et al., 2004). Several field studies show that the stem water content of woody plants decreased during acclimation (Ashworth et al., 1993; Pagter et al., 2011b) which was also observed for ‘Chandos Beauty’ (Ouyang et al., 2019a). However, in this study, controlled acclimation did not lead to any decrease of the stem water content of ‘Chandos Beauty’ (Figure 2B) which may be attributed to the relative short cold treatment. Dehydrins are correlated to both dehydration and cold hardiness (Hanin et al., 2011). Both rose cultivars had a stable stem water content, thus changes in the expression of *RhDHN5* must be induced by a temperature response.

*RhDHN5* gradually increased during the different steps of cold acclimation and strongly correlates with cold hardiness levels for both cultivars (Figure 4 and Table 1). This is consistent with the results of seasonal acclimation in roses (Ouyang et al., 2019a) and of bark tissues in both evergreen and deciduous peach genotypes (Arora and Wisniewski, 1996; Wisniewski et al., 1996; Artlip et al., 1997). As our first cold acclimation step also includes a transition to short days, we cannot exclude a photoperiodic response as well. However, in hybrid aspen and silver birch, transcript levels of dehydrin showed only a limited response to photoperiod but increased strongly under decreasing temperature (Welling et al., 2002; Puhakainen, 2004). Also in peach bark, *ppdhn1* did not respond to 3 weeks of short days at 20°C (Gusta et al., 2005). The abrupt decrease of *RhDHN5* transcript levels after 1 day of deacclimation (22/18°C, 8 h) suggests that *RhDHN5* may be linked to freezing avoidance by promoting supercooling. A similar sharp decrease in protein abundance of a 105-kD dehydrin was observed within 1 day of deacclimation in *Rhododendron viscosum* var. serrulatum and was assumed to be associated with the freezing avoidance strategy (supercooling) (Kalberer et al., 2007). It is noteworthy that our stem samples are including both bark tissues and xylem tissues. The dehydrin protein PCA60 (encoded by *ppdh1*, homolog of *RhDHN5*) in peach was freely located in cytoplasm, plastids, and nucleus of bark cells and xylem ray parenchyma cells (Wisniewski et al., 1999). Under cold stress, PCA60 was assumed to bind to ice to prevent water to migrate to ice crystals and therefore the proteins that accumulated within xylem ray parenchyma may inhibit intracellular ice nucleation and promote supercooling in the cells (Wisniewski et al., 1999). Supercooling in xylem tissue may also be a strategy to prevent the damage of cellular dehydration observed in several woody-plant species (Gusta et al., 1983). Freezing tolerance and freezing avoidance (supercooling) are not mutually exclusive and may both contribute to the development of cold hardiness of woody plants (Wisniewski and Ashworth, 1986; Wisniewski et al., 2018).

A high number of solutes is another acclimation strategy in cold tolerance. Starch-to-sugar interconversion is commonly observed in woody plants during cold acclimation and deacclimation (Sauter, 1988). In this study total soluble sugars increased during cold acclimation, especially for ‘Chandos Beauty’ where they also strongly correlated to LT_50_. Low temperature and corresponding low respiration rates as well as translocation of sugars from senescing leaves might explain this net carbon gain in the stems (Levitt, 1980; Kalberer et al., 2006). Enzymes of the starch catabolic pathway responded to cold acclimation. *RhBAM3* was induced by low temperatures, as is also reported for other species (Elle and Sauter, 2000; Kaplan et al., 2006). For ‘Dagmar Hastrup,’ a decrease to 2°C induced a transient up-regulation of *RhBAM3* but no further obvious increase was found at sub-zero temperatures (Figure 5A). In contrast, temperatures of 2°C induced *RhBAM3* expression for ‘Chandos Beauty,’ which increased further at −3°C (Figure 5B). The up-regulation of *RhBAM3* during cold acclimation was not clearly present in the field study (Ouyang et al., 2019a). Here we identified a transient expression pattern induced by low temperature which makes it difficult to detect in the longer time-span samples. *RhBAM3* (Acc No. MH249079, Supplementary Table S4), was extracted according to *VcBMY* of blueberry (*Vaccinium corymbosum* L.) (Supplementary Table S2). *VcBMY* was reported to be associated with cold hardiness and its closest homolog in *Arabidopsis* is *AtBAM3* (identity 71%). In *Arabidopsis*, several *BAM* gene family members were isolated but *BAM3* (also called *BMY8*) is the only cold shock induced amylase gene (Maruyama et al., 2009). *AtBAM3* or its homolog in other plants was reported as playing a dominant role in hydrolyzing starch (Scheidig et al., 2002; Kaplan and Guy, 2005). *PtrBAM1*, a close homolog of *AtBAM3* isolated from trifoliate orange (*Poncirus trifoliata*), can be directly regulated by CBF and contributes to cold hardiness by regulating sugar content (Peng et al., 2014). In our study, the up-regulation of *RhBAM3* may also directly regulate the accumulation of soluble sugars (Figures 3B, 5A,B). Maltose is the direct breakdown product of starch by chloroplast-localized BAM which is then converted into glucose in the cytosol by DPE2 (disproportionating enzyme 2) (Kaplan et al., 2006). Only for ‘Dagmar Hastrup’, this conversion was found as *RhDPE2* was upregulated by both 2 and −3°C (Figure 5C) and this was also shown during seasonal acclimation (Ouyang et al., 2019a). This is also visible at metabolic level, with increasing hexose levels observed under cold acclimation. For ‘Chandos Beauty’, an accumulation of maltose levels might be expected, which by itself can protect membranes and proteins in the stroma (Kaplan et al., 2006).

Glucose and fructose are converted into Glc-6-P and Fru-6-P, which are precursors of sucrose biosynthesis by HXK and FRK. In *Arabidopsis*, the transcript levels of *HXK* and *FRK* were abundant during cold acclimation, thus contributing to the early increase of Glc-6-P and Fru-6-P (Kaplan et al., 2007). In tea plant (*Camellia sinensis*), *CsHXK3* is up-regulated during cold acclimation and is related to sugar-signaling (Yue et al., 2015). However, although our *RhHXK1* (MH249074, Supplementary Table S4) is the homolog sequence of *CsHXK3* (KJ489422, Supplementary Table S2), the transcript levels were rather suppressed. Similarly, the expression of *RhFRK4* was also down-regulated.

The conversion of hexoses to sucrose enhanced sucrose levels at −3°C for ‘Chandos Beauty’ (Figures 3C,D). In contrast, the overall sucrose levels of ‘Dagmar Hastrup’ were lower and decreased at low temperature (Figure 3C). SPS is the key enzyme in sucrose synthesis in both photosynthetic and non-photosynthetic tissues (Dali et al., 1992; Gutiorrez-Miceli et al., 2002). In both rose cultivars, however, *RhSPS1* transcripts decreased under cold acclimation (Figure 5D). Changes in soluble sugars do not strongly correlate with the transcript abundances during cold acclimation. This may be due to the posttranslational modifications that changes the stability of the related enzymes (Toroser and Huber, 1997). The function of SUS is more for sucrose degradation than sucrose synthesis and its activity correlates positively to starch synthesis (Dochlert, 1990; Dali et al., 1992). In both rose cultivars, *RhSUS* transcript levels were down-regulated when temperature decreased (Figure 5E) which is also reported in a previous field study during November–January (Ouyang et al., 2019a). Furthermore, invertase activity can result in cleavage of sucrose into glucose and fructose. No up-regulation of *RhINV2* under cold acclimation is present for ‘Chandos Beauty.’

It is well-established that high sugar amounts and especially RFOs are correlated to cold hardiness in different organs (shoots, leaf, and buds) of woody species (Hinesley et al., 1992; Ögren, 1997; Imanishi et al., 1998; Palonen et al., 2000; Pagter et al., 2008; Van Labeke and Volckaert, 2010; Trischuk et al., 2014). Raffinose and stachyose accumulate prominently for both cultivars during acclimation and the levels correlated with LT_50_ values, indicating their importance in cold acclimation (Table 1). RFOs, although present in smaller amounts at cellular level, are the most effective inhibitors to prevent crystallization of sucrose (Koster and Leopold, 1988). Raffinose biosynthesis was faster for the less cold-hardy ‘Chandos Beauty,’ where moderate temperatures (13/6°C) with short days already induced biosynthesis, although accumulation was strongly induced by low temperatures (2°C) and sub-zero temperatures (−3°C) (Figure 3E). Stachyose reached higher levels for ‘Chandos Beauty’ at −3°C, but overall stachyose levels of both cultivars were low (Figure 3F). RFOs (raffinose and stachyose) accumulation was also clearly observed in the seasonal study (Ouyang et al., 2019a).

Biosynthesis of RFOs starts from galactinol, which is synthesized from UDP-galactose and myo-inositol through galactinol synthase. Myo-inositol is produced from Glc6P by myo-inositol phosphate synthase (MIPS) and UDP-galactose is formed from D-galactoside by GK. Despite the increase of RFOs in roses under cold stress, *RhMIPS* was clearly downregulated by both moderate (13/6°C) and lower temperatures (2 and −3°C) (Figure 5I) confirming earlier field observations (Ouyang et al., 2019a).

In ‘Dagmar Hastrup’ only, *RhGK* was induced by both 2 and −3°C. The increasing UDP-galactose levels thus correlate with the accumulation of raffinose (Figure 5J). *Raffinose synthase* (*RS*) encodes the enzyme of the rate-limiting step in raffinose biosynthesis and its regulation precedes the biosynthesis of raffinose (Kaplan et al., 2007). The expression of *RhRS6* was induced early for ‘Dagmar Hastrup’ when temperatures decreased from 13/6 to 2°C, corresponding to the rapid increase of raffinose and stachyose during this period (Figures 3E,F, 5K). For ‘Chandos Beauty’ significant up-regulation started later and was strongly induced by sub-zero temperatures (Figure 5K). *RS* up-regulation under low temperature was also reported in *Arabidopsis*, grape and tea plant (Kaplan et al., 2007; Yue et al., 2015; Chai et al., 2019). *RhGK* and *RhRS6* showed a clearer up-regulation pattern under low temperatures in the controlled-climate study compared to the field study (Ouyang et al., 2019a).

The reversibility of the cold response during deacclimation on the gene expression level was strongly present in the cold associated genes (*RhDPE2* for ‘Dagmar Hastrup,’ *RhBAM3* and *RhDHN5* for both cultivars). While gene expression data during deacclimation have rarely been reported in stems/bark of woody plants, the decrease of dehydrins and metabolites such as RFOs has been reported in several studies (Kalberer et al., 2006; Pagter et al., 2011b; Shin et al., 2015).

This reversibility of the cold response was also found in the up-regulation of *RhSUS*, *RhSPS1*, *RhHXK1*, *RhFRK4*, *RhINV2*, and *RhMIPS* for ‘Chandos Beauty’ and *RhSUS*, *RhSPS1*, *RhFRK4*, and *RhMIPS* for ‘Dagmar Hastrup’ at 1 and/or 2 weeks after the start of the deacclimation, which is related to bud burst and shooting as more carbon in the sink organs is needed (Figures 5D–H). Most transcripts were more strongly enhanced during deacclimation for the moderately cold-hardy ‘Chandos Beauty’ than for the more cold-hardy ‘Dagmar Hastrup.’ This response is also found in to the mean time to bud break (MTB) observed for both cultivars: bud break and shooting in ‘Chandos Beauty’ was earlier than in ‘Dagmar Hastrup’ (Figure 1).

## Conclusion

*Dehydrin* (*RhDHN5*) and carbohydrate metabolism were both associated with the development of cold hardiness. The up regulation of *RhDHN5* during acclimation is mainly triggered by low temperatures as stem water content remained stable. Soluble sugars accumulated with up-regulated of *RhBAM3* during cold acclimation. Raffinose and stachyose accumulated greatly during cold acclimation, but the associated genes (*RhRS6* and *RhGK*) of their biosynthesis showed genotype specific up-regulation. Although similar cold hardiness levels were reached in both rose genotypes, differences in carbohydrate metabolism and related gene expression were present. The different regulation may be linked to their distinct genetic background and indicates different cold adaptation strategies in rose genotypes. Deacclimation was fast in both genotypes. The lack of endodormancy in both cultivars may increase the risk for shoot damage in these new deacclimated shoots when temperature drops again.

## Data Availability Statement

All datasets generated for this study are included in the article/Supplementary Material.

## Author Contributions

LO and M-CV planned the experiments. LO conducted the experiments and analyzed all the data. LO, ED and M-CV interpreted the data. LO wrote the manuscript. M-CV and LL critically revised the manuscript. All authors read and approved the manuscript.

## Conflict of Interest

The authors declare that the research was conducted in the absence of any commercial or financial relationships that could be construed as a potential conflict of interest.

## Abbreviations

BAM/BMY: β-amylase
DHN: dehydrin
DPE2: disproportionating enzyme 2
EL: Electrolyte leakage
FRK: fructokinase
GK: galactokinase
HXK: hexokinase
INV: invertase
*I*_*t*_: index of injury
LT_50_: the temperature causing 50% of index of injury
MIPS: myo-inositol-phosphate synthase
PPFD: photosynthetic photon flux density
RFOs: raffinose family oligosaccharides
Rh: Rosa × hybrida
RS: raffinose synthase
SPS: sucrose phosphate synthase
SUS: sucrose synthase
USDA: the United States Department of Agriculture.
