# Supplementary material for: Cold Acclimation and Deacclimation of Two Garden Rose Cultivars Under Controlled Daylength and Temperature
Source: Front Plant Sci. 2020 Mar 24;11:327. doi: 10.3389/fpls.2020.00327 (PMC7105705; doi:10.3389/fpls.2020.00327)
Supplement: Supplementary file 1 [file Data_Sheet_1.docx]

**Article title**: Cold acclimation and deacclimation of two garden rose cultivars under controlled daylength and temperature

**Journal**: Frontiers in Plant Science

**Authors**: Lin Ouyang, Leen Leus, Ellen De Keyser and Marie-Christine Van Labeke

**Corresponding author**: Lin Ouyang, Institute of Urban Agriculture, Chinese Academy of Agricultural Sciences; Email: linouyang1101@outlook.com

**Supplementary tables** Table S1-S4, Reference to this file

**Table S1** Nanodrop data of RNA samples of ‘Dagmar Hastrup’ and ‘Chandos Beauty’.

| Genotypes | Sampling dates | Biological replicates | Nucleic acid concentration (ng/ul) | A260/A280 | A260/A230 |
| --- | --- | --- | --- | --- | --- |
| 'Dagmar Hastrup' | 19th Dec 16 | 1 | 58.91 | 1.94 | 1.40 |
| 'Dagmar Hastrup' | 19th Dec 16 | 2 | 59.56 | 1.99 | 1.39 |
| 'Dagmar Hastrup' | 19th Dec 16 | 3 | 70.41 | 2.02 | 2.14 |
| 'Chandos Beauty' | 19th Dec 16 | 1 | 191.19 | 2.05 | 2.06 |
| 'Chandos Beauty' | 19th Dec 16 | 2 | 243.14 | 2.03 | 2.04 |
| 'Chandos Beauty' | 19th Dec 16 | 3 | 151.16 | 2.06 | 1.99 |
| 'Dagmar Hastrup' | 20th Dec 16 | 1 | 104.33 | 2.06 | 1.81 |
| 'Dagmar Hastrup' | 20th Dec 16 | 2 | 99.95 | 2.04 | 1.76 |
| 'Dagmar Hastrup' | 20th Dec 16 | 3 | 88.35 | 2.06 | 1.68 |
| 'Chandos Beauty' | 20th Dec 16 | 1 | 170.70 | 2.06 | 1.98 |
| 'Chandos Beauty' | 20th Dec 16 | 2 | 192.65 | 2.00 | 2.03 |
| 'Chandos Beauty' | 20th Dec 16 | 3 | 192.34 | 2.02 | 2.00 |
| 'Dagmar Hastrup' | 2nd Jan 17 | 1 | 68.28 | 2.05 | 1.48 |
| 'Dagmar Hastrup' | 2nd Jan 17 | 2 | 75.78 | 2.08 | 1.69 |
| 'Dagmar Hastrup' | 2nd Jan 17 | 3 | 87.73 | 2.01 | 1.65 |
| 'Chandos Beauty' | 2nd Jan 17 | 1 | 152.62 | 2.07 | 1.90 |
| 'Chandos Beauty' | 2nd Jan 17 | 2 | 162.89 | 2.01 | 1.98 |
| 'Chandos Beauty' | 2nd Jan 17 | 3 | 172.00 | 2.03 | 2.02 |
| 'Dagmar Hastrup' | 16th Jan 17 | 1 | 83.23 | 2.08 | 1.68 |
| 'Dagmar Hastrup' | 16th Jan 17 | 2 | 141.18 | 2.04 | 1.90 |
| 'Dagmar Hastrup' | 16th Jan 17 | 3 | 86.96 | 2.06 | 1.71 |
| 'Chandos Beauty' | 16th Jan 17 | 1 | 209.39 | 2.00 | 1.99 |
| 'Chandos Beauty' | 16th Jan 17 | 2 | 191.51 | 2.03 | 1.76 |
| 'Chandos Beauty' | 16th Jan 17 | 3 | 172.27 | 2.03 | 2.00 |
| 'Dagmar Hastrup' | 17th Jan 17 | 1 | 77.33 | 1.96 | 1.56 |
| 'Dagmar Hastrup' | 17th Jan 17 | 2 | 91.38 | 2.06 | 1.74 |
| 'Dagmar Hastrup' | 17th Jan 17 | 3 | 70.88 | 1.97 | 1.60 |
| 'Chandos Beauty' | 17th Jan 17 | 1 | 257.76 | 2.02 | 2.07 |
| 'Chandos Beauty' | 17th Jan 17 | 2 | 200.04 | 2.03 | 1.95 |
| 'Chandos Beauty' | 17th Jan 17 | 3 | 205.37 | 2.02 | 1.99 |
| 'Dagmar Hastrup' | 23th Jan 17 | 1 | 101.07 | 2.07 | 1.77 |
| 'Dagmar Hastrup' | 23th Jan 17 | 2 | 80.85 | 2.02 | 1.65 |
| 'Dagmar Hastrup' | 23th Jan 17 | 3 | 93.63 | 2.00 | 1.79 |
| 'Chandos Beauty' | 23th Jan 17 | 1 | 213.05 | 2.04 | 2.10 |
| 'Chandos Beauty' | 23th Jan 17 | 2 | 209.58 | 2.02 | 2.08 |
| 'Chandos Beauty' | 23th Jan 17 | 3 | 196.26 | 2.04 | 1.96 |
| 'Dagmar Hastrup' | 30th Jan 17 | 1 | 88.84 | 2.01 | 1.56 |
| 'Dagmar Hastrup' | 30th Jan 17 | 2 | 94.31 | 1.97 | 1.53 |
| 'Dagmar Hastrup' | 30th Jan 17 | 3 | 77.44 | 2.07 | 1.64 |
| 'Chandos Beauty' | 30th Jan 17 | 1 | 132.99 | 2.04 | 1.95 |
| 'Chandos Beauty' | 30th Jan 17 | 2 | 200.87 | 2.03 | 1.96 |
| 'Chandos Beauty' | 30th Jan 17 | 3 | 257.14 | 2.03 | 2.06 |
| 'Dagmar Hastrup' | 31th Jan 17 | 1 | 104.63 | 2.03 | 1.84 |
| 'Dagmar Hastrup' | 31th Jan 17 | 2 | 87.46 | 2.04 | 1.78 |
| 'Dagmar Hastrup' | 31th Jan 17 | 3 | 116.78 | 2.07 | 1.79 |
| 'Chandos Beauty' | 31th Jan 17 | 1 | 121.59 | 2.09 | 1.82 |
| 'Chandos Beauty' | 31th Jan 17 | 2 | 227.02 | 2.05 | 2.02 |
| 'Chandos Beauty' | 31th Jan 17 | 3 | 206.17 | 2.04 | 2.10 |
| 'Dagmar Hastrup' | 6th Feb 17 | 1 | 162.91 | 2.02 | 2.05 |
| 'Dagmar Hastrup' | 6th Feb 17 | 2 | 102.81 | 2.01 | 1.81 |
| 'Dagmar Hastrup' | 6th Feb 17 | 3 | 106.50 | 2.00 | 1.71 |
| 'Chandos Beauty' | 6th Feb 17 | 1 | 207.33 | 2.04 | 2.03 |
| 'Chandos Beauty' | 6th Feb 17 | 2 | 235.26 | 2.02 | 2.10 |
| 'Chandos Beauty' | 6th Feb 17 | 3 | 212.85 | 2.00 | 1.97 |
| 'Dagmar Hastrup' | 13th Feb 17 | 1 | 68.50 | 2.02 | 1.75 |
| 'Dagmar Hastrup' | 13th Feb 17 | 2 | 71.48 | 2.03 | 1.61 |
| 'Dagmar Hastrup' | 13th Feb 17 | 3 | 82.64 | 2.06 | 1.71 |
| 'Chandos Beauty' | 13th Feb 17 | 1 | 182.93 | 2.01 | 2.04 |
| 'Chandos Beauty' | 13th Feb 17 | 2 | 248.91 | 2.01 | 2.11 |
| 'Chandos Beauty' | 13th Feb 17 | 3 | 221.96 | 2.02 | 2.09 |
| 'Dagmar Hastrup' | 14th Feb 17 | 1 | 61.68 | 2.02 | 2.21 |
| 'Dagmar Hastrup' | 14th Feb 17 | 2 | 70.13 | 2.01 | 1.53 |
| 'Dagmar Hastrup' | 14th Feb 17 | 3 | 80.66 | 2.02 | 1.68 |
| 'Chandos Beauty' | 14th Feb 17 | 1 | 221.96 | 2.01 | 2.02 |
| 'Chandos Beauty' | 14th Feb 17 | 2 | 133.92 | 2.01 | 1.74 |
| 'Chandos Beauty' | 14th Feb 17 | 3 | 230.9 | 2.04 | 2.23 |
| 'Dagmar Hastrup' | 20th Feb 17 | 1 | 66.1 | 1.95 | 1.71 |
| 'Dagmar Hastrup' | 20th Feb 17 | 2 | 67.73 | 1.98 | 1.34 |
| 'Dagmar Hastrup' | 20th Feb 17 | 3 | 79.96 | 1.98 | 1.83 |
| 'Chandos Beauty' | 20th Feb 17 | 1 | 194.60 | 2.03 | 2.03 |
| 'Chandos Beauty' | 20th Feb 17 | 2 | 194.24 | 2.00 | 1.93 |
| 'Chandos Beauty' | 20th Feb 17 | 3 | 159.08 | 2.00 | 1.81 |
| 'Dagmar Hastrup' | 27th Feb 17 | 1 | 50.13 | 2.12 | 1.80 |
| 'Dagmar Hastrup' | 27th Feb 17 | 2 | 107.73 | 1.98 | 1.74 |
| 'Dagmar Hastrup' | 27th Feb 17 | 3 | 46.44 | 2.00 | 2.17 |
| 'Chandos Beauty' | 27th Feb 17 | 1 | 93.99 | 2.01 | 1.89 |
| 'Chandos Beauty' | 27th Feb 17 | 2 | 61.80 | 1.98 | 1.49 |
| 'Chandos Beauty' | 27th Feb 17 | 3 | 121.33 | 2.00 | 1.9 |

**Table S2** Candidate genes in other species used to identify the putative homologue and to be isolated from the *Rosa*. spp transcriptome database.

| Genes in roses | Functional annotation | Species | Acc. No. | Reference |
| --- | --- | --- | --- | --- |
| *RhDHN5* | dehydrin | *Prunus persica* | U34809 | Artlip et al. (1997) |
| *RhSPS1* | sucrose-phosphate synthase | *Camellia sinensis* | KF696388 | Yue et al. (2015) |
| *RhSUS** | sucrose synthase | *Camellia sinensis* | KF921302 | Yue et al. (2015) |
| *RhINV2** | invertase | *Camellia sinensis* | KP053402 | Yue et al. (2015) |
| *RhHXK1* | hexokinase | *Camellia sinensis* | KJ489422 | Yue et al. (2015) |
| *RhFRK4* | fructokinase | *Camellia sinensis* | KF696390 | Yue et al. (2015) |
| *RhRS6** | raffinose synthesis | *Camellia sinensis* | KP162174 | Yue et al. (2015) |
| *RhMIPS** | inositol-3-phosphate synthase | *Camellia sinensis* | KP053396 | Yue et al. (2015) |
| *RhGK** | galactokinase | *Camellia sinensis* | KF703739 | Yue et al. (2015) |
| *RhBAM3* | beta-amylase | *Vaccinium corymbosum* | JQ911593 | Lee et al. (2012) |
| *RhDPE2** | 4-alpha-glucanotransferase activity | *Camellia sinensis* | KP053399 | Yue et al. (2015) |

*Genes isolated from our previous article (Ouyang et al., 2019)

**Table S3** Sequence homology (BLASTx, default settings) of the isolated *Rosa*. spp gene fragments with orthologues in other species.

| Acc. N° | Species | Description | Query coverage (%) | Max ident | E-value |
| --- | --- | --- | --- | --- | --- |
| *RhDHN5** |  |  |  |  |  |
| PRQ47518.1 | *Rosa chinensis* | putative dehydrin | 100 | 0.94 | 1.00E-26 |
| XP_024180019.1 | *Rosa chinensis* | PREDICTED: cold-shock protein CS120-like isoform X1 | 100 | 0.94 | 2.00E-26 |
| XP_004287863.1 | *Fragaria vesca* subsp. *vesca* | PREDICTED: dehydrin Xero 2 | 50 | 0.71 | 3.00E-18 |
| *RhBAM3** |  |  |  |  |  |
| XP_024172552.1 | *Rosa chinensis* | PREDICTED: beta-amylase 3 (chloroplastic) | 99 | 0.99 | 0 |
| XP_004300297.1 | *Fragaria vesca* subsp. *vesca* | PREDICTED: beta-amylase 3 (chloroplastic) | 99 | 0.95 | 0 |
| XP_007209867.1 | *Prunus persica* | PREDICTED: beta-amylase 3 (chloroplastic) | 99 | 0.86 | 0 |
| *RhDPE2** |  |  |  |  |  |
| XP_024187658.1 | *Rosa chinensis* | PREDICTED: 4-alpha-glucanotransferase DPE2 | 99 | 0.99 | 0 |
| XP_004309467.1 | *Fragaria vesca* subsp. *vesca* | PREDICTED: 4-alpha-glucanotransferase DPE2 | 99 | 0.92 | 0 |
| XP_020423512.1 | *Prunus persica* | PREDICTED: 4-alpha-glucanotransferase DPE2 | 99 | 0.84 | 0 |
| *RhSPS1* |  |  |  |  |  |
| XP_024179865.1 | *Rosa chinensis* | PREDICTED: probable sucrose-phosphate synthase 1 | 99 | 0.99 | 0 |
| XP_004287272.1 | *Fragaria vesca* subsp. *vesca* | PREDICTED: probable sucrose-phosphate synthase 1 | 99 | 0.96 | 0 |
| XP_007204296.1 | *Prunus persica* | PREDICTED: probable sucrose-phosphate synthase 1 | 99 | 0.91 | 0 |
| *RhSUS** |  |  |  |  |  |
| XP_024180521.1 | *Rosa chinensis* | PREDICTED: sucrose synthase | 99 | 1.00 | 0 |
| XP_004287669.1 | *Fragaria vesca* subsp. *vesca* | PREDICTED: sucrose synthase | 99 | 0.96 | 0 |
| XP_007204649.1 | *Prunus persica* | PREDICTED: sucrose synthase | 99 | 0.89 | 0 |
| *RhINV2** |  |  |  |  |  |
| XP_024170683.1 | *Rosa chinensis* | PREDICTED: acid beta-fructofuranosidase 2, vacuolar | 99 | 0.98 | 0 |
| XP_004298661.1 | *Fragaria vesca* subsp. *vesca* | PREDICTED: acid beta-fructofuranosidase-like | 99 | 0.92 | 0 |
| XP_007210314.1 | *Prunus persica* | PREDICTED: acid beta-fructofuranosidase 2, vacuolar | 99 | 0.78 | 0 |
| *RhHXK1* |  |  |  |  |  |
| XP_024180097.1 | *Rosa chinensis* | PREDICTED: hexokinase-1-like | 82 | 1.00 | 0 |
| XP_004287480.1 | *Fragaria vesca* subsp. *vesca* | PREDICTED: hexokinase-1 | 82 | 0.97 | 0 |
| XP_007203872.1 | *Prunus persica* | PREDICTED: hexokinase-1 | 82 | 0.94 | 0 |
| *RhFRK4* |  |  |  |  |  |
| XP_024196842.1 | *Rosa chinensis* | PREDICTED: probable fructokinase-4 | 89 | 0.99 | 0 |
| XP_004309811.1 | *Fragaria vesca* subsp. *vesca* | PREDICTED: probable fructokinase-4 | 89 | 0.94 | 0 |
| XP_007222617.1 | *Prunus persica* | PREDICTED: probable fructokinase-4 | 91 | 0.89 | 0 |
| *RhGK** |  |  |  |  |  |
| XP_024197777.1 | *Rosa chinensis* | PREDICTED: galactokinase | 99 | 0.99 | 0 |
| XP_004295062.1 | *Fragaria vesca* subsp. *vesca* | PREDICTED: galactokinase | 99 | 0.97 | 0 |
| XP_007223314.1 | *Prunus persica* | PREDICTED: galactokinase | 99 | 0.86 | 0 |
| *RhMIPS** |  |  |  |  |  |
| XP_024182441.1 | *Rosa chinensis* | PREDICTED: inositol-3-phosphate synthase | 99 | 1.00 | 0 |
| XP_004288224.1 | *Fragaria vesca* subsp. *vesca* | PREDICTED: inositol-3-phosphate synthase | 99 | 0.98 | 0 |
| XP_007205055.1 | *Prunus persica* | PREDICTED: inositol-3-phosphate synthase | 99 | 0.96 | 0 |
| *RhRS6** |  |  |  |  |  |
| XP_024181138.1 | *Rosa chinensis* | PREDICTED: probable galactinol--sucrose galactosyltransferase 6 | 99 | 0.99 | 0 |
| XP_004287282.1 | *Fragaria vesca* subsp. *vesca* | PREDICTED: probable galactinol--sucrose Galactosyltransferase 6 | 91 | 0.9 | 0 |
| XP_007204875.1 | *Prunus persica* | PREDICTED: probable galactinol--sucrose Galactosyltransferase 6 | 91 | 0.8 | 0 |

* Genes isolated from our previous article (Ouyang et al., 2019)

**Table S4** List of RT-qPCR primer sequences and product size for *Rosa*. spp target gene fragments and reference genes. PCR efficiencies were determined using LinRegPCR (Ruijter et al., 2009).

| Genes | Acc. No. | F or R | Primer sequence 5'-3' | Amplicon size (bp) | PCR efficiencies |
| --- | --- | --- | --- | --- | --- |
| *RhDHN5** | MH249069 | F | GGTCACAAGGACGATCCCTA | 86 | 1.856 |
|  |  | R | CCCTTATGCTCTTGGTGCTC |  |  |
| *RhSPS1* | MH249071 | F | CCCAAGCCCTCAGGTATTTA | 103 | 1.891 |
|  |  | R | CCGAGCAACCCTTCATAATC |  |  |
| *RhSUS** | MH249072 | F | AGACCCTTCTCACTGGGACA | 142 | 1.852 |
|  |  | R | GCGATCAAGGTTGGAGACA |  |  |
| *RhINV2** | MH249073 | F | TCTGTGGCAACTGATGTTGTT | 130 | 1.873 |
|  |  | R | TTGTTCGTCCACCTTGAGC |  |  |
| *RhHXK1* | MH249074 | F | TGGAGTGGGGTAACTTTCGT | 140 | 1.912 |
|  |  | R | TCTGCGCACAATCTCTCCTA |  |  |
| *RhFRK4* | MH249075 | F | TTCCGTGGATCTGTGGAAG | 135 | 1.893 |
|  |  | R | TTCCCTCAATCTTGGCTCAT |  |  |
| *RhRS6** | MH249076 | F | CATTAGTGGCGGACCTGTTT | 84 | 1.904 |
|  |  | R | CCGTCCGGCAATACTATCTT |  |  |
| *RhMIPS** | MH249077 | F | ACCCTGATTTCATTGCTGCT | 133 | 1.901 |
|  |  | R | ACCACCTTGTCCACTTTGCT |  |  |
| *RhGK** | MH249078 | F | GCCTCAAGACCAAGTTCCAC | 88 | 1.824 |
|  |  | R | TGCTCTCCGATCAAGTTCAC |  |  |
| *RhBAM3** | MH249079 | F | AAGTTCCCAGGAATTGGAGAA | 132 | 1.820 |
|  |  | R | CTGATTGTACTGGCCGGAAT |  |  |
| *RhDPE2** | MH249080 | F | ACCAAGATACCTTTTCCGTTCA | 80 | 1.874 |
|  |  | R | AGAACATTGGGATTGCAACA |  |  |
| *RhPG^#^* | EC586265.1 | F | GCCAAAGTCATCTTGGCTTC | 101 | 1.874 |
|  |  | R | CCACTCCAAGGAGCTCAGAC |  |  |
| *RhRPS18c^#^* | BI977264.1 | F | ATCTCGAGCGGTTGAAGAAG | 97 | 1.870 |
|  |  | R | TGCGACCAGTAGTCTTGGTG |  |  |

* Primers were designed in our previous article (Ouyang et al., 2019); ^#^primers are based on Pipino. L. (Pipino, 2011).

**References**

Artlip, T. S., Callahan, A. M., Bassett, C. L., and Wisniewski, M. E. (1997). Seasonal expression of a dehydrin gene in sibling deciduous and evergreen genotypes of peach (*Prunus persica* [L.] Batsch). *Plant Mol. Biol.* 33, 61–70. doi:10.1023/A:1005787909506.

Lee, J. H., Yu, D. J., Kim, S. J., Choi, D., and Lee, H. J. (2012). Intraspecies differences in cold hardiness, carbohydrate content and β-amylase gene expression of *Vaccinium corymbosum* during cold acclimation and deacclimation. *Tree Physiol.* 32, 1533–1540. doi:10.1093/treephys/tps102.

Ouyang, L., Leus, L., De Keyser, E., and Van Labeke, M.-C. (2019). Seasonal changes in cold hardiness and carbohydrate metabolism in four garden rose cultivars. *J. Plant Physiol.* 232, 188–199. doi:10.1016/j.jplph.2018.12.001.

Pipino, L. (2011). Improving seed production efficiency for hybrid rose breeding.[PhD’s thesis], [Ghent]:Ghent University.

Razavi, F.(2012). Molecular and physiological responses to drought stress in Fragaria sp. [PhD’s thesis, [Ghent]: Ghent University.

Ruijter, J. M., Ramakers, C., Hoogaars, W. M. H., Karlen, Y., Bakker, O., Van den hoff, M. J. B., et al. (2009). Amplification efficiency: linking baseline and bias in the analysis of quantitative PCR data. *Nucleic Acids Res.* 37, e45–e45. doi:10.1093/nar/gkp045.

Yue, C., Cao, H. L., Wang, L., Zhou, Y. H., Huang, Y. T., Hao, X. Y., et al. (2015). Effects of cold acclimation on sugar metabolism and sugar-related gene expression in tea plant during the winter season. *Plant Mol. Biol.* 88, 591–608. doi:10.1007/s11103-015-0345-7.
