# Supplementary material for: Cold Acclimation and Deacclimation of Two Garden Rose Cultivars Under Controlled Daylength and Temperature
Source: Front Plant Sci. 2020 Mar 24;11:327. doi: 10.3389/fpls.2020.00327 (PMC7105705; doi:10.3389/fpls.2020.00327)
Supplement: Supplementary file 2 [file Data_Sheet_2.docx]

**Article title**: Cold acclimation and deacclimation of two garden rose cultivars under controlled daylength and temperature

**Journal**: Frontiers in Plant Science

**Authors**: Lin Ouyang, Leen Leus, Ellen De Keyser and Marie-Christine Van Labeke

**Corresponding author**: Lin Ouyang, Institute of Urban Agriculture, Chinese Academy of Agricultural Sciences; Email: linouyang1101@outlook.com

**Supplementary figures** Figures S1-S3


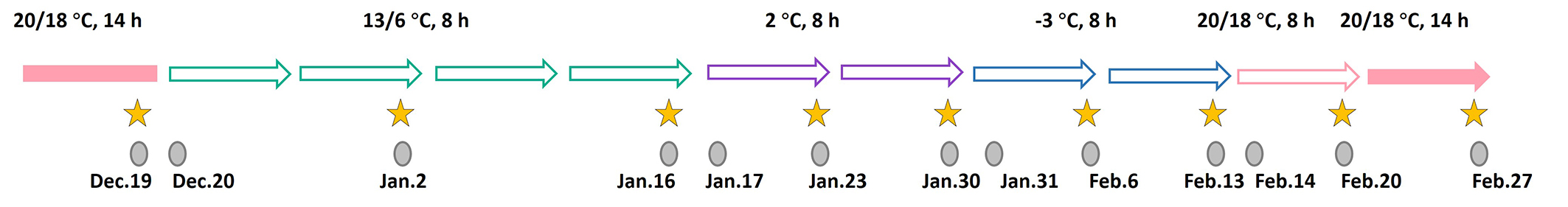


**Figure S1** Sampling points for the acclimation/deacclimation experiment (Exp.2). Each arrow indicates a one-week period. Stars indicate the sampling points for determination of cold hardiness, analysis of stem water content and stem carbohydrate content. Circles indicate the sampling points for gene expression analysis.


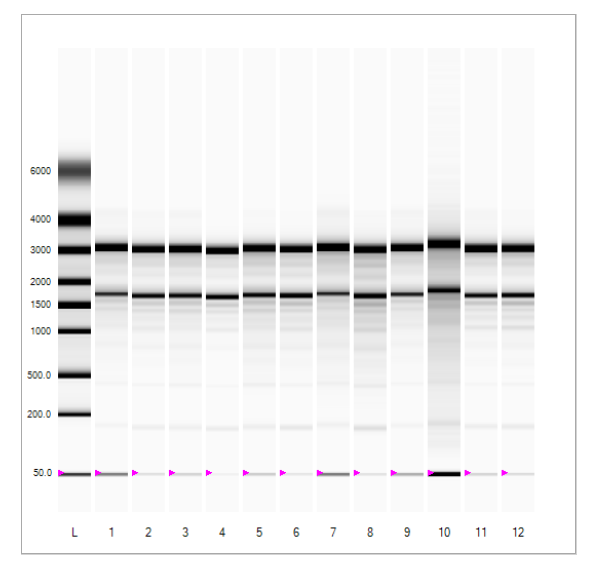


**CB (1)**

**6 Feb.**

**CB (1)**

**17 Jan.**

**DH (1)**

**14 Feb.**

**DH (1)**

**31 Jan.**

**DH (1)**

**27 Feb.**

**Ladder**

**DH (2)**

**16 Dec.**

**DH (1)**

**16 Dec.**

**CB (1)**

**20 Dec.**

**DH (3)**

**27 Feb.**

**CB (3)**

**14 Feb.**

**CB (1)**

**27 Feb.**

**CB (3)**

**27 Feb.**

**Figure S2** Virtual gel image (Experion) of DNase treated RNA samples. Estimated sizes of marker bands (lane L) are presented in kilodaltons (kDa). CB=‘Chandos Beauty’, DH=‘Dagmar Hastrup’; the sampling dates are presented as month_date. The numbers between brackets indicate the biological replicate of the cultivar under corresponding sampling dates.

**CB (1)**

**20 Dec.**

**DH (1)**

**31 Jan.**

**CB (3)**

**14 Feb.**

**CB (1)**

**27 Feb.**

**DH (1)**

**16 Dec.**

**Ladder**


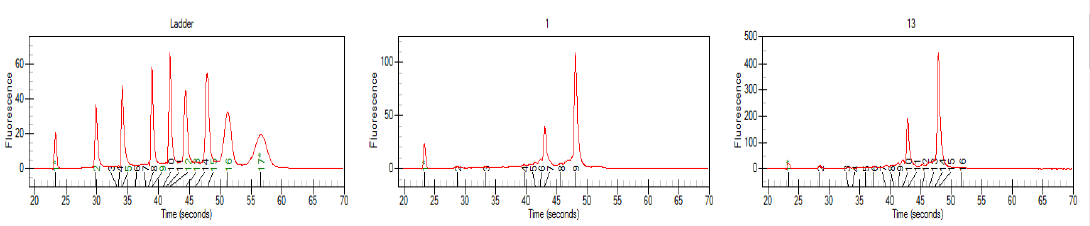


**DH (3)**

**27 Feb.**

**DH (1)**

**14 Feb.**

**CB (1)**

**17 Jan.**

**DH (2)**

**16 Jan.**


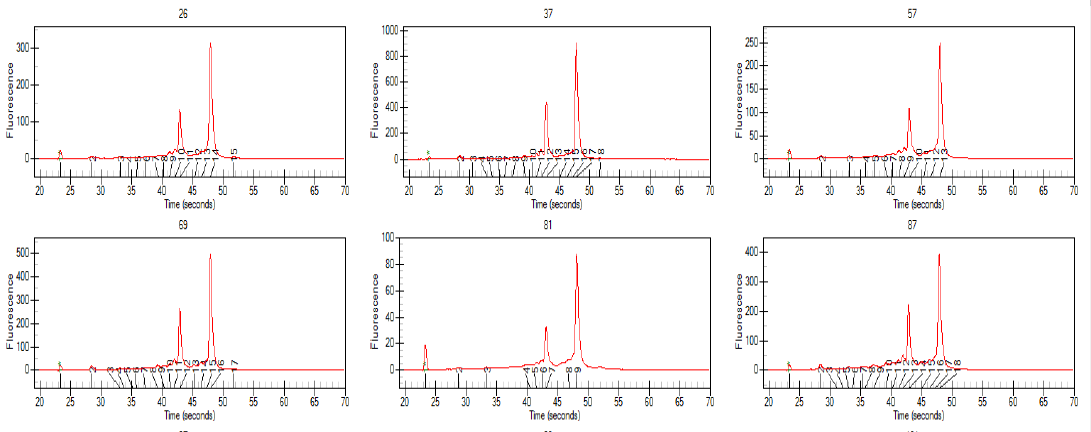


**CB (1)**

**6 Feb.**

*
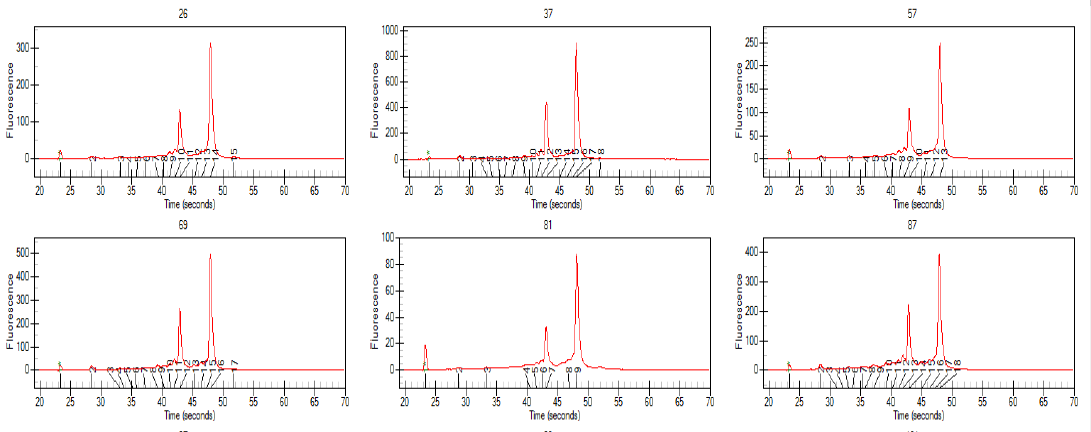
*

**DH (1)**

**27 Feb.**


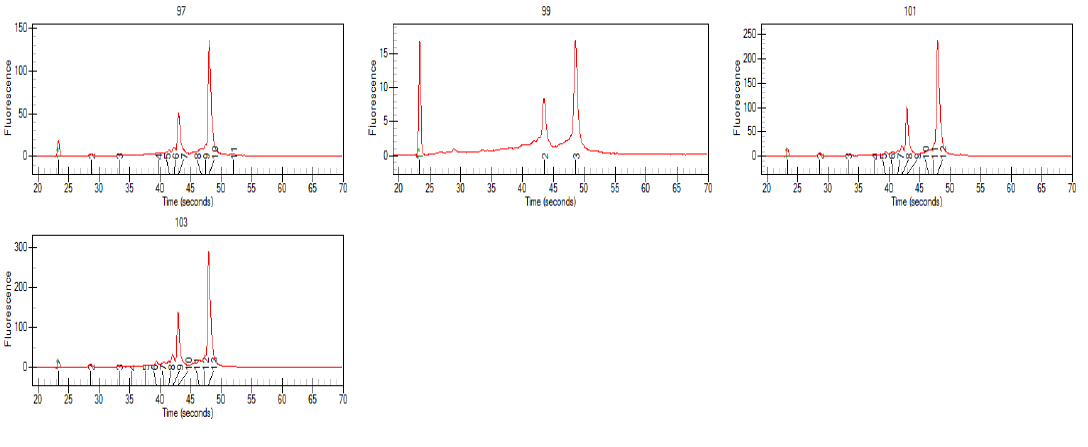


**CB (3)**

**27 Feb.**

*
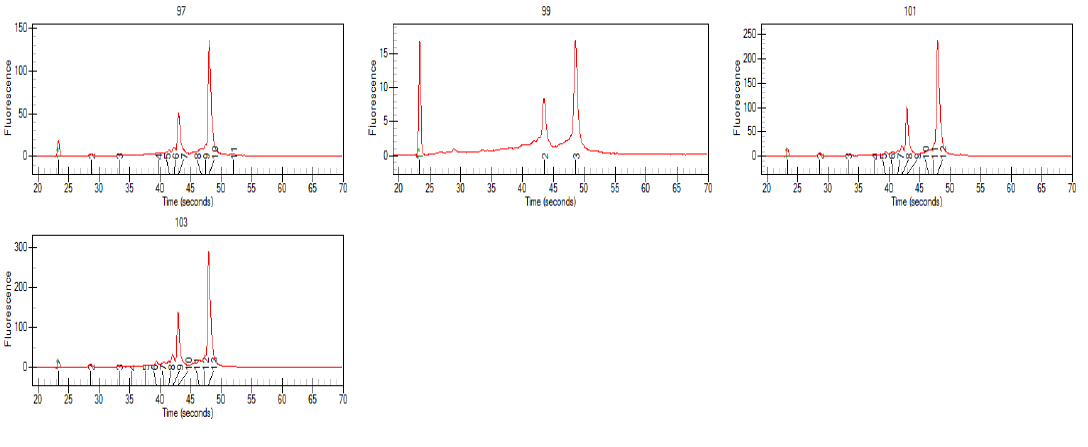
*

**Figure S3** Electropherograms (Experion) of DNase treated RNA sample. Estimated sizes of marker bands (lane L) are presented in kilodaltons (kDa). CB=‘Chandos Beauty’, DH=‘Dagmar Hastrup’; the sampling dates are presented as month_date. The numbers between brackets indicate the biological replicate of the cultivar under corresponding sampling dates.
